# New half-discrete Hilbert inequalities for three variables

**DOI:** 10.1186/s13660-017-1594-6

**Published:** 2018-01-03

**Authors:** Tserendorj Batbold, Laith E. Azar

**Affiliations:** 1 Department of Mathematics, National University of Mongolia, Ulaanbaatar, 14201 Mongolia; 2 Department of Mathematics, Al al-Bayt University, P.O. Box 130095, Mafraq, Jordan

**Keywords:** 26D15, Hilbert inequality, half-discrete inequality, the best possible constant, equivalent form

## Abstract

In this paper, we obtain two new half-discrete Hilbert inequalities for three variables. The obtained inequalities are with the best constant factor. Moreover, we give their equivalent forms.

## Introduction

Suppose that $p>1$, $\frac{1}{p}+\frac{1}{q}=1$, and *f* and *g* are nonnegative functions such that $f\in L_{p} ( \mathbb{R}_{+} )$ and $g\in L_{q} (\mathbb{R}_{+} )$. Then we have the inequalities

1 $$\begin{aligned}& \int_{0}^{\infty} \int_{0}^{\infty}\frac{f(x)g(y)}{x+y}\,dx\,dy< \frac{\pi}{\sin({\pi}/{p})} \biggl( \int_{0}^{\infty}f^{p}(x)\,dx \biggr)^{\frac{1}{p}} \biggl( \int_{0}^{\infty}g^{q}(y)\,dy \biggr)^{\frac{1}{q}}, \end{aligned}$$

2 $$\begin{aligned}& \int_{0}^{\infty} \biggl( \int_{0}^{\infty}\frac{f(x)}{x+y}\,dx \biggr)^{p}\,dy< \biggl(\frac{\pi}{\sin({\pi}/{p}) } \biggr)^{p} \int_{0}^{\infty}f^{p}(x)\,dx, \end{aligned}$$

provided that the integrals on the right-hand side in both inequalities are positive. The constant factors $\frac{\pi}{\sin({\pi}/{p}) }$ and $(\frac{\pi}{\sin({\pi}/{p})} )^{p}$ are the best possible in (1) and (2), respectively. Moreover, (1) and (2) are equivalent. Inequality (1) was first studied by D. Hilbert at the end of the 19th century, and hence, in his honor, it is referred to as the Hilbert inequality.

The corresponding discrete forms of inequalities (1) and (2) for two nonnegative sequences of real numbers $\{ a_{m} \} $ and $\{ b_{n} \} $ are given as

3 $$\begin{aligned}& \sum_{m=1}^{\infty}\sum _{n=1}^{\infty}\frac{a_{m}b_{n}}{m+n}< \frac{\pi}{\sin({\pi}/{p})} \Biggl(\sum_{m=1}^{\infty}a_{m}^{p} \Biggr)^{\frac{1}{p}} \Biggl(\sum_{n=1}^{\infty}b_{n}^{q} \Biggr)^{\frac{1}{q}}, \end{aligned}$$

4 $$\begin{aligned}& \sum_{n=1}^{\infty} \Biggl( \sum _{m=1}^{\infty}\dfrac{a_{m}}{ m+n} \Biggr) ^{p}< \biggl(\frac{\pi}{\sin({\pi}/{p})} \biggr)^{p}\sum _{m=1}^{\infty}a_{m}^{p}, \end{aligned}$$

where $\{ a_{m} \} \in\ell_{p}$ and $\{ b_{n} \} \in\ell_{q}$. The constant factors $\frac{\pi}{\sin({\pi}/{p}) }$ and $(\frac{\pi}{\sin({\pi}/{p})} )^{p}$ are also the best possible. Inequalities (3) and (4) are equivalent (see [1]).

During the last twenty years, both inequalities (1) and (3) and their equivalent forms were generalized and extended in many different ways (see [2–13]).

Yang [14] introduced the following half-discrete Hilbert inequality:

5 $$ \begin{aligned} [b]& \int_{0}^{\infty}f(x)\sum_{n=1}^{\infty} \frac{a_{n}}{ ( x+n ) ^{\lambda}}\,dx \\ &\quad< B(\lambda_{1},\lambda_{2}) \biggl( \int_{0}^{\infty}x^{p(1-\lambda_{1})-1}f^{p}(x)\,dx \biggr) ^{\frac {1}{p}} \Biggl( \sum_{n=1}^{\infty}n^{q(1-\lambda_{2})-1}a_{n}^{q} \Biggr) ^{\frac{1}{q}},\end{aligned} $$

where, $\lambda_{1},\lambda_{2}>0$, $\lambda_{1}+\lambda_{2}=\lambda$, $0<\lambda_{1}<1$, and the constant $B(\lambda_{1},\lambda_{2})$ is the best possible. In particular, for $\lambda=1$ and $\lambda_{1}=\frac {1}{q}$, $\lambda_{2}=\frac{1}{p}$, the previous inequality reduces to

6 $$ \int_{0}^{\infty}f(x)\sum_{n=1}^{\infty} \frac{a_{n}}{x+n}\,dx< \frac{\pi}{\sin(\pi/q)} \biggl( \int_{0}^{\infty}f^{p}(x)\,dx \biggr)^{\frac {1}{p}} \Biggl(\sum_{n=1}^{\infty}a_{n}^{q} \Biggr)^{\frac{1}{q}}. $$

For extensions and other half-discrete Hilbert inequalities, we refer the reader to [15–18].

Very recently, a new form of the Hilbert inequality for three variables was obtained in [19]:

7 $$ \begin{aligned}[b]& \int_{0}^{\infty} \int_{0}^{\infty} \int_{0}^{\infty}\frac{f(x,y)g(z)}{ ( x+y+z ) ^{\lambda}}\,dx\,dy\,dz \\ &\quad\leq C \biggl( \int_{0}^{\infty} \int_{0}^{\infty} ( x+y ) ^{2p-\lambda-p\gamma-2}f^{p}(x,y)\,dx\,dy \biggr) ^{\frac{1}{p}} \\ &\qquad\times \biggl( \int_{0}^{\infty}z^{q+q\gamma-\lambda -1}g^{q}(z)\,dz \biggr) ^{\frac{1}{q}},\end{aligned} $$

where $\lambda>0$, $\gamma\in ( \frac{-\lambda}{p},\frac {\lambda}{q} ) $, $f(x,y)$ is a nonnegative function on $( 0,\infty ) \times ( 0,\infty ) $, $g(z)>0$ on $( 0,\infty ) $, and the constant $C=B ( \frac{\lambda}{p}+\gamma,\frac{ \lambda}{q}-\gamma ) $ is the best possible.

In this paper, we obtain, under some suitable conditions, two new half-discrete inequalities with the same best constant factor as in (7). Moreover, we give two equivalent forms for established inequalities. All the obtained equivalent forms are also sharp.

## Preliminaries and lemmas

As it is well known, the gamma function $\Gamma(\theta)$ and beta function $B ( \mu,\nu ) $ are defined respectively by the improper integrals

$$\begin{gathered} \Gamma(\theta)= \int_{0}^{\infty}t^{\theta-1}e^{-t}\,dt, \quad\theta>0, \\B ( \mu,\nu ) = \int_{0}^{\infty}\frac{t^{\mu-1}}{ ( t+1 ) ^{\mu+\nu}}\,dt,\quad\mu, \nu>0.\end{gathered} $$

Using the definition of the gamma function, we may write

8 $$ \frac{1}{ ( x+y ) ^{\lambda}}=\frac{1}{\Gamma ( \lambda ) } \int_{0}^{\infty}t^{\lambda-1}e^{- ( x+y ) t}\,dt. $$

To prove our main results, we need some lemmas. As we will see, their proofs are simple and based on the famous Hölder inequality for both integrals and sums and the fact that we can estimate the sum of a decreasing function by an integral. The first lemma (Lemma 2.1) is given in [19] and the second one (Lemma 2.2) is also given in [15]. For completeness, we give proofs of these two lemmas.

### Lemma 2.1

([19])

*If* $p>1$, $\frac{1}{p}+\frac{1}{q}=1 $, $t>0$, $\alpha<\frac{2}{q}$, *and* $f(x,y)$ *is a nonnegative function defined and integrable on* $( 0,\infty ) \times ( 0,\infty ) $, *then*

9 $$ \begin{aligned}[b]& \biggl( \int_{0}^{\infty} \int_{0}^{\infty }e^{-t(x+y)}f(x,y)\,dx\,dy \biggr) ^{p} \\ &\quad\leq \bigl( t^{\alpha q-2}\Gamma(2-\alpha q) \bigr) ^{\frac{p}{q}} \int_{0}^{\infty} \int_{0}^{\infty } (x+y ) ^{\alpha p}e^{-t(x+y)}f^{p}(x,y)\,dx\,dy.\end{aligned} $$

### Proof

By the Hölder inequality we obtain

10 $$ \begin{aligned}[b]& \biggl( \int_{0}^{\infty} \int_{0}^{\infty }e^{-t(x+y)}f(x,y)\,dx\,dy \biggr) ^{p} \\ &\quad= \biggl( \int_{0}^{\infty } \int_{0}^{\infty} \bigl\{ ( x+y ) ^{-\alpha}e^{-\frac{t(x+y)}{q}} \bigr\} \bigl\{ ( x+y ) ^{\alpha}e^{-\frac {t(x+y)}{p}}f(x,y)\bigr\} \,dx\,dy\biggr) ^{p} \\ &\quad\leq \biggl( \int_{0}^{\infty} \int_{0}^{\infty} ( x+y ) ^{-\alpha q}e^{-t(x+y)}\,dx\,dy \biggr) ^{\frac{p}{q}} \\ &\quad\quad\times \int_{0}^{\infty} \int_{0}^{\infty} ( x+y ) ^{\alpha p}e^{-t(x+y)}f^{p}(x,y)\,dx\,dy.\end{aligned} $$

We compute the first integral on the right-hand side of (10) by using the substitutions $y=ux$ and $x=\frac{v}{t(u+1)}$:

11 $$ \begin{aligned}[b] \int_{0}^{\infty} \int_{0}^{\infty} ( x+y ) ^{-\alpha q}e^{-t(x+y)}\,dx\,dy&= \int_{0}^{\infty} ( 1+u ) ^{-\alpha q} \int_{0}^{\infty}x^{1-\alpha q}e^{-tx(1+u)}\,dx\,du \\ &=t^{\alpha q-2} \int_{0}^{\infty} ( 1+u ) ^{-2}\,du \int_{0}^{\infty}v^{1-\alpha q}e^{-v}\,dv \\ &=t^{\alpha q-2}\Gamma (2-\alpha q).\end{aligned} $$

Substituting (11) into (10), we get (9). □

### Lemma 2.2

([15])

*Let* $p>1$, $\frac{1}{p}+\frac {1}{q}=1$, *and* $a_{n}>0$. *Then*, *for* $t>0$ *and* $0\leq \theta<\frac{1}{p}$, *we have*

$$ \sum_{n=1}^{\infty}e^{-nt}a_{n} \leq t^{\theta-\frac{1}{p}}\Gamma (1-\theta p)^{\frac{1}{p}} \Biggl( \sum _{n=1}^{\infty}n^{\theta q}e^{-nt}a_{n}^{q} \Biggr) ^{\frac{1}{q}}. $$

### Proof

Using the Hölder inequality, we get

$$\begin{aligned} \sum_{n=1}^{\infty}e^{-nt}a_{n} =& \sum_{n=1}^{\infty} \bigl\{ n^{-\theta}e^{-\frac{nt}{p}} \bigr\} \bigl\{ n^{\theta}e^{-\frac{nt}{q}}a_{n} \bigr\} \\ \leq& \Biggl( \sum_{n=1}^{\infty}n^{-\theta p}e^{-nt} \Biggr) ^{\frac {1}{p}} \Biggl( \sum_{n=1}^{\infty}n^{\theta q}e^{-nt}a_{n}^{q} \Biggr) ^{\frac{1}{q}} \\ \leq& \biggl( \int_{0}^{\infty}x^{-\theta p}e^{-tx}\,dx \biggr) ^{\frac {1}{p}} \Biggl( \sum_{n=1}^{\infty}n^{\theta q}e^{-nt}a_{n}^{q} \Biggr) ^{\frac {1}{q}} \\ =&t^{\theta-\frac{1}{p}}\Gamma(1-\theta p)^{\frac{1}{p}} \Biggl( \sum _{n=1}^{\infty}n^{\theta q}e^{-nt}a_{n}^{q} \Biggr)^{\frac{1}{q}}. \end{aligned}$$

The lemma is proved. □

### Lemma 2.3

*Let* $p>1$, $\frac{1}{p}+\frac{1}{q}=1$, *and* $a_{n, m}>0$. *Then*, *for* $t>0$ *and* $0\leq\theta<\frac{1}{p}$, *we have*

$$ \Biggl( \sum_{m=1}^{\infty}\sum _{n=1}^{\infty}e^{-(n+m)t}a_{n,m} \Biggr) ^{p}\leq \bigl( t^{\theta q-2}\Gamma(2-\theta q) \bigr) ^{\frac{p}{q}} \sum_{m=1}^{\infty}\sum _{n=1}^{\infty} ( m+n ) ^{\theta p}e^{-(n+m)t}a_{n,m}^{p}. $$

### Proof

The proof follows the lines of the proof of Lemma 2.2. Namely, applying Hölder inequality, we have

$$\begin{aligned} & \Biggl(\sum_{m=1}^{\infty}\sum _{n=1}^{\infty}e^{-(n+m)t}a_{n,m} \Biggr) ^{p} \\ &\quad= \Biggl( \sum_{m=1}^{\infty}\sum _{n=1}^{\infty} \bigl\{ (m+n)^{-\theta }e^{-\frac{(n+m)t}{q}} \bigr\} \bigl\{ (m+n)^{\theta}e^{-\frac {(n+m)t}{q}}a_{n,m} \bigr\} \Biggr)^{p} \\ &\quad\leq \Biggl( \sum_{m=1}^{\infty}\sum _{n=1}^{\infty}(m+n)^{-\theta q}e^{-(n+m)t} \Biggr) ^{\frac{p}{q}}\sum_{m=1}^{\infty}\sum _{n=1}^{\infty }(m+n)^{\theta p}e^{-(n+m)t}a_{n,m}^{p} \\ &\quad\leq \biggl( \int_{0}^{\infty} \int_{0}^{\infty} ( x+y ) ^{-\theta q}e^{-(x+y)t}\,dx\,dy \biggr) ^{\frac{p}{q}}\sum_{m=1}^{\infty }\sum _{n=1}^{\infty}(m+n)^{\theta p}e^{-(n+m)t}a_{n,m}^{p} \\ &\quad= \bigl( t^{\theta q-2}\Gamma(2-\theta q) \bigr) ^{\frac{p}{q}}\sum _{m=1}^{\infty}\sum_{n=1}^{\infty}(m+n)^{\theta p}e^{-(n+m)t}a_{n,m}^{p}.\end{aligned} $$

The lemma is proved. □

Similarly, we obtain the following lemma.

### Lemma 2.4

*Let* $p>1$, $\frac{1}{p}+\frac{1}{q}=1$, *and* $f\geq0$. *Then*, *for* $t>0$ *and* $\alpha>-\frac{1}{p}$, *we have*

$$ \biggl( \int_{0}^{\infty}e^{-tx}f(x)\,dx \biggr) ^{q}\leq \bigl( t^{-1-\alpha p}\Gamma(\alpha p+1) \bigr) ^{\frac{q}{p}} \int _{0}^{\infty}x^{-\alpha q}e^{-tx}f^{q}(x)\,dx. $$

## Main results

In this section, we give two new half-discrete versions of inequality (7). Both obtained inequalities are with the best constant factor expressed in terms of the beta function.

### Theorem 3.1

*Let* $p>1$, $\frac{1}{p}+\frac{1}{q}=1$, $\lambda>0$, $\max\{\frac{\lambda }{q}-1,-\frac{\lambda}{p}\}\leq \gamma<\frac{\lambda}{q}$, *and* $a_{n}>0$. *Suppose that* $f(x,y)$ *is a nonnegative function defined on* $( 0,\infty ) \times ( 0,\infty ) $. *If* $\int_{0}^{\infty }\int_{0}^{\infty} ( x+y ) ^{2p-\lambda-p\gamma -2}f^{p}(x,y)\,dx\,dy<\infty$ *and* $\sum_{n=1}^{\infty }n^{\gamma q+q-\lambda-1}a_{n}^{q}<\infty$, *then*

12 $$ \begin{aligned}[b]& \int_{0}^{\infty} \int_{0}^{\infty }f(x,y)\sum _{n=1}^{\infty}\frac{a_{n}}{ ( x+y+n ) ^{\lambda}}\,dx\,dy\\ &\quad=\sum _{n=1}^{\infty}a_{n} \int_{0}^{\infty } \int_{0}^{\infty}\frac{f(x,y)}{ ( x+y+n ) ^{\lambda }}\,dx\,dy \\ &\quad\leq C \biggl( \int_{0}^{\infty} \int_{0}^{\infty} ( x+y ) ^{2p-\lambda-p\gamma-2}f^{p}(x,y)\,dx\,dy \biggr)^{\frac{1}{p}} \Biggl( \sum_{n=1}^{\infty}n^{\gamma q+q-\lambda-1}a_{n}^{q} \Biggr) ^{\frac{1}{q}},\end{aligned} $$

*where the constant* $C=B ( \frac{\lambda}{q}-\gamma,\frac{ \lambda}{p}+\gamma ) $ *is the best possible*. *In particular*:

1. *for* $\lambda=1$, $\gamma=0$, *and* $p=q=2$, *we have*
  $$\begin{aligned} & \int_{0}^{\infty} \int_{0}^{\infty }f(x,y)\sum _{n=1}^{\infty}\frac{a_{n}}{x+y+n}\,dx\,dy \\ &\quad\leq\pi \biggl( \int_{0}^{\infty} \int_{0}^{\infty} ( x+y ) f^{2}(x,y)\,dx\,dy \biggr)^{\frac{1}{2}} \Biggl( \sum_{n=1}^{\infty }a_{n}^{2} \Biggr)^{\frac{1}{2}};\end{aligned} $$
2. *for* $\lambda=2$, $\gamma=0$, *and* $p=q=2$, *we have*
  $$\begin{aligned} & \int_{0}^{\infty} \int_{0}^{\infty }f(x,y)\sum _{n=1}^{\infty}\frac{a_{n}}{ ( x+y+n ) ^{2}} \,dx\,dy \leq \biggl( \int_{0}^{\infty} \int_{0}^{\infty }f^{2}(x,y)\,dx\,dy \biggr) ^{\frac{1}{2}} \Biggl( \sum_{n=1}^{\infty} \frac{ a_{n}^{2}}{n} \Biggr) ^{\frac{1}{2}}.\end{aligned} $$

### Proof

Using (8) and applying the Hölder inequality, we have

13 $$ \begin{aligned}[b] I&= \int_{0}^{\infty} \int_{0}^{\infty }f(x,y)\sum _{n=1}^{\infty}\frac{a_{n}}{ ( x+y+n ) ^{\lambda}}\,dx\,dy \\ &=\frac{1}{\Gamma ( \lambda ) } \int_{0}^{\infty} \int_{0}^{\infty}f(x,y)\sum _{n=1}^{\infty }a_{n} \biggl( \int_{0}^{\infty}t^{\lambda-1}e^{- ( x+y+n ) t}\,dt \biggr)\,dx\,dy \\ &=\frac{1}{\Gamma ( \lambda ) } \int_{0}^{\infty} \biggl( t^{\frac{\lambda-1}{p}+\gamma} \int_{0}^{\infty } \int_{0}^{\infty}e^{-xt-yt}f(x,y)\,dx\,dy \biggr) \Biggl( t^{\frac{\lambda-1}{q}-\gamma}\sum_{n=1}^{\infty}e^{-nt}a_{n} \Biggr)\,dt \\ &\leq\frac{1}{\Gamma ( \lambda ) } \biggl( \int_{0}^{\infty}t^{\lambda-1+p\gamma} \biggl( \int_{0}^{\infty} \int_{0}^{\infty }e^{-xt-yt}f(x,y)\,dx\,dy \biggr) ^{p}\,dt \biggr) ^{\frac{1}{p}} \\ &\quad\times \Biggl( \int_{0}^{\infty}t^{\lambda-1-q\gamma} \Biggl( \sum _{n=1}^{\infty}e^{-nt}a_{n} \Biggr) ^{q}\,dt \Biggr) ^{\frac{1}{q}}.\end{aligned} $$

By Lemma 2.1 and Lemma 2.2 we obtain respectively

$$\begin{aligned} & \biggl( \int_{0}^{\infty} \int_{0}^{\infty }e^{-(x+y)t}f(x,y)\,dx\,dy \biggr) ^{p} \\ &\quad\leq \bigl[ t^{\alpha q-2}\Gamma(2-\alpha q) \bigr] ^{\frac{p}{q}} \int_{0}^{\infty} \int_{0}^{\infty } ( x+y ) ^{\alpha p}e^{-(x+y)t}f^{p}(x,y)\,dx\,dy\end{aligned} $$

and

$$ \Biggl( \sum_{n=1}^{\infty}e^{-nt}a_{n} \Biggr) ^{q}\leq t^{\theta q-q+1}\Gamma (1-\theta r)^{\frac{q}{p}}\sum _{n=1}^{\infty}n^{\theta q}e^{-nt}a_{n}^{q}. $$

Putting these two inequalities into (13), we get

$$\begin{aligned} I&\leq\frac{\Gamma(2-\alpha q)^{\frac{1}{q}}\Gamma(1-\theta p)^{\frac {1}{p}}}{\Gamma ( \lambda ) } \\ &\quad\times \biggl( \int_{0}^{\infty } \int_{0}^{\infty}f^{p}(x,y) \biggl( \int_{0}^{\infty} ( x+y ) ^{\alpha p}t^{p\gamma+\lambda+\alpha p-2p+1}e^{-xt-yt}\,dt \biggr) \,dx\,dy \biggr) ^{\frac{1}{p}} \\ &\quad\times \Biggl( \sum_{n=1}^{\infty}n^{\theta q}a_{n}^{q} \int_{0}^{\infty}t^{\lambda+\theta q-q-\gamma q}e^{-nt}\,dt \Biggr) ^{\frac{1}{q}}.\end{aligned} $$

Since

$$ \int_{0}^{\infty}t^{\lambda+p\gamma+p\alpha -2p+1}e^{-(x+y)t}\,dt= ( x+y ) ^{-\lambda-p\gamma-p\alpha +2p-2}\Gamma ( \lambda+p\gamma+p\alpha-2p+2 ) $$

and

$$ \int_{0}^{\infty}t^{\lambda-q\gamma+q\theta -q}e^{-nt}\,dt=n^{-1-\lambda+q\gamma-q\theta+q} \Gamma ( \lambda -q\gamma+q\theta-q+1 ) , $$

it follows that

$$ I\leq C_{\alpha, \theta} \biggl( \int_{0}^{\infty} \int_{0}^{\infty} ( x+y ) ^{2p-\lambda-p\gamma-2}f^{p}(x,y)\,dx\,dy \biggr) ^{\frac{1}{p}} \Biggl( \sum_{n=1}^{\infty}n^{\gamma q+q-\lambda -1}a_{n}^{q} \Biggr) ^{\frac{1}{q}}, $$

where

$$C_{\alpha, \theta}=\frac{\Gamma(2-\alpha q)^{\frac{1}{q}}\Gamma (1-\theta p)^{\frac{1}{p}}\Gamma ( \lambda+p\gamma+p\alpha -2p+2 )^{\frac{1}{p}}\Gamma ( \lambda -q\gamma-q\theta-q+1 )^{\frac{1}{q}}}{\Gamma(\lambda)}. $$

Now, setting $\alpha=\frac{2p-p\gamma-\lambda}{pq}$ and $\theta =\frac{q\gamma-\lambda+q}{pq}$, we get inequality (12) with the constant $C_{\frac{2p-p\gamma-\lambda}{pq}, \frac{q\gamma-\lambda +q}{pq}}=C$. It remains to show that the constant factor *C* in (12) is the best possible. For sufficiently small positive *ε*, define the function $f_{\varepsilon}(x,y)=0$ on $( 0,1 ) \times ( 0,1 ) $ and $f_{\varepsilon}(x,y)=(x+y)^{\frac {\lambda -\varepsilon}{p}+\gamma-2}$ on $[ 1,\infty ) \times [ 1,\infty ) $ and the sequence $\tilde{a}_{n}=n^{\frac{\lambda -\varepsilon}{q}-\gamma-1}$ ($n\geq1 $). Suppose that the constant *C* is not the best possible. Then there exist $0< K< C$ such that

14 $$\begin{aligned} I&\leq K \biggl( \int_{0}^{\infty} \int_{0}^{\infty} ( x+y ) ^{2p-\lambda-p\gamma-2}f_{\varepsilon}^{p}(x,y)\,dx\,dy \biggr) ^{\frac{1}{p}} \Biggl( \sum_{n=1}^{\infty}n^{\gamma q+q-\lambda-1}\tilde{a}_{n}^{q} \Biggr) ^{\frac{1}{q}} \\ &=K \biggl( \int_{1}^{\infty} \int_{1}^{\infty} ( x+y ) ^{-\varepsilon-2}\,dx\,dy \biggr) ^{\frac{1}{p}} \Biggl( \sum_{n=1}^{\infty}n^{-\varepsilon-1} \Biggr)^{\frac{1}{q}} \\ &\leq K \biggl( \int_{1}^{\infty}x^{-1-\varepsilon} \int_{\frac{1}{x} }^{\infty} ( 1+u ) ^{-\varepsilon-2}\,du\,dx \biggr) ^{\frac{1}{p} } \biggl( 1+ \int_{1}^{\infty}u^{-1-\varepsilon}\,du \biggr) ^{\frac {1}{q}} \\ &=\frac{K}{ ( \varepsilon+1 ) ^{\frac{1}{p}}} \biggl( \int_{1}^{\infty}\frac{1}{(x+1)^{1+\varepsilon}}\,dx \biggr) ^{\frac{1}{p}} \biggl( 1+\frac{1}{\varepsilon} \biggr) ^{\frac{1}{q}} \\ &= \frac{K}{\varepsilon}\frac{(1+\varepsilon)^{\frac{1}{q}}}{2^{\frac{\varepsilon }{p}}(1+\varepsilon)^{\frac{1}{p}}}. \end{aligned}$$

On the other hand, estimating the left-hand side, we find (set $u=z(x+y)$)

15 $$ \begin{aligned}[b] I&= \int_{1}^{\infty} \int_{1}^{\infty }\sum_{n=1}^{\infty} \frac{f_{\varepsilon}(x,y)\tilde{a}_{n}}{ ( x+y+n ) ^{\lambda}}\,dx\,dy \\ &= \int_{1}^{\infty } \int_{1}^{\infty}(x+y)^{\frac{\lambda-\varepsilon}{p}+\gamma -2}\sum _{n=1}^{\infty}\frac{n^{\frac{\lambda-\varepsilon}{q}-\gamma-1}}{ ( x+y+n ) ^{\lambda}}\,dx\,dy \\ &\geq \int_{1}^{\infty} \int_{1}^{\infty}(x+y)^{\frac{\lambda -\varepsilon}{p}+\gamma-2} \biggl( \int_{1}^{\infty}\frac{u^{\frac{\lambda-\varepsilon}{q}-\gamma-1}}{ ( x+y+u ) ^{\lambda}}\,du \biggr)\,dx\,dy \\ &= \int_{1}^{\infty} \int_{1}^{\infty}(x+y)^{-\varepsilon -2} \biggl( \int_{\frac{1}{x+y}}^{\infty}\frac{z^{\frac{\lambda -\varepsilon}{q}-\gamma-1}}{ ( z+1 ) ^{\lambda}}\,du \biggr) \,dx\,dy \\ &= \int_{1}^{\infty} \int_{1}^{\infty}(x+y)^{-\varepsilon-2} \biggl[ \int_{0}^{\infty}\frac{z^{\frac{\lambda-\varepsilon}{q}-\gamma-1}}{ ( z+1 ) ^{\lambda}}\,du- \int_{0}^{\frac{1}{x+y}}\frac{z^{\frac{\lambda-\varepsilon}{q}-\gamma-1}}{ ( z+1 ) ^{\lambda}}\,dz \biggr] \,dx\,dy \\ &=\frac{B ( \frac{\lambda-\varepsilon}{q}-\gamma,\frac{\lambda }{p}+\gamma+\frac{\varepsilon}{p} ) }{\varepsilon2^{\varepsilon }(1+\varepsilon)}- \int_{1}^{\infty} \int_{1}^{\infty }(x+y)^{-\varepsilon-2} \int_{0}^{\frac{1}{x+y}}\frac{z^{\frac{\lambda-\varepsilon}{q}-\gamma-1}}{ ( z+1 ) ^{\lambda }}\,dz\,dx\,dy \\ &\geq\frac{B ( \frac{\lambda-\varepsilon}{q}-\gamma,\frac {\lambda}{p}+\gamma+\frac{\varepsilon}{p} ) }{\varepsilon2^{\varepsilon }(1+\varepsilon)}- \int_{1}^{\infty} \int_{1}^{\infty }(x+y)^{-\varepsilon-2} \int_{0}^{\frac{1}{x+y}}z^{\frac{\lambda -\varepsilon}{q}-\gamma-1}\,dz\,dx\,dy \\ &=\frac{B ( \frac{\lambda-\varepsilon}{q}-\gamma,\frac{\lambda }{p}+\gamma+\frac{\varepsilon}{p} ) }{\varepsilon2^{\varepsilon }(1+\varepsilon)}-O(1).\end{aligned} $$

Obviously, as $\varepsilon\rightarrow0^{+}$, from (14) and (15) we obtain a contradiction. Therefore, the proof of the theorem is completed. □

### Theorem 3.2

*Let* $p>1$, $\frac{1}{p}+\frac{1}{q}=1$, $\lambda>0$, $-\frac{\lambda}{p}<\gamma<\min(2-\frac{\lambda }{p},\frac{\lambda}{q})$, *and* $a_{n,m}>0$. *Suppose that* $f(x)$ *is a nonnegative function defined on* $( 0,\infty ) $ *and a double sequence* ${a_{n,m}>0}$.

*If* $\sum_{m=1}^{\infty}\sum_{n=1}^{\infty} ( m+n )^{2p-\lambda-p\gamma-2}a_{n,m}^{p}<\infty$ *and* $\int_{0}^{\infty}x^{q\gamma+q-\lambda-1}f^{q}(x)\,dx<\infty$, *then*

16 $$ \begin{aligned}[b]&\sum_{m=1}^{\infty}\sum _{n=1}^{\infty }a_{n,m} \int_{0}^{\infty}\frac{f(x)}{ ( x+m+n ) ^{\lambda}}\,dx\\ &\quad= \int_{0}^{\infty}f(x) \Biggl( \sum _{m=1}^{\infty }\sum_{n=1}^{\infty} \frac{a_{n,m}}{ ( x+m+n ) ^{\lambda}} \Biggr) \,dx \\ &\quad\leq C \Biggl( \sum_{m=1}^{\infty}\sum _{n=1}^{\infty} ( m+n ) ^{2p-\lambda-p\gamma-2}a_{n,m}^{p} \Biggr) ^{\frac {1}{p}} \biggl( \int_{0}^{\infty}x^{\gamma q+q-\lambda-1}f^{q}(x)\,dx \biggr) ^{\frac{1}{q}},\end{aligned} $$

*where the constant* *C* *is the best possible*. *In particular*:

1. *for* $\lambda=1$, $\gamma=0$, *and* $p=q=2$, *we have*
  $$\begin{aligned} & \int_{0}^{\infty}f(x) \Biggl( \sum _{m=1}^{\infty }\sum_{n=1}^{\infty} \frac{a_{n,m}}{x+m+n} \Biggr) \,dx \\ &\quad\leq\pi \Biggl( \sum_{m=1}^{\infty}\sum _{n=1}^{\infty} ( m+n ) a_{n,m}^{2} \Biggr) ^{\frac{1}{2}} \biggl( \int_{0}^{\infty }f^{2}(x)\,dx \biggr) ^{\frac{1}{2}}.\end{aligned} $$
2. *for* $\lambda=2$, $\gamma=0$, *and* $p=q=2$, *we have*
  $$\begin{aligned} & \int_{0}^{\infty}f(x) \Biggl( \sum _{m=1}^{\infty }\sum_{n=1}^{\infty} \frac{a_{n,m}}{ ( x+m+n ) ^{2}} \Biggr) \,dx \\ &\quad\leq \Biggl( \sum_{m=1}^{\infty}\sum _{n=1}^{\infty }a_{n,m}^{2} \Biggr) ^{\frac{1}{2}} \biggl( \int_{0}^{\infty}\frac{f^{2}(x)}{x}\,dx \biggr) ^{\frac{1}{2}}.\end{aligned} $$

### Proof

Using (8) and applying the Hölder inequality, we have

17 $$ \begin{aligned}[b]J&= \int_{0}^{\infty}f(x) \Biggl( \sum _{m=1}^{\infty }\sum_{n=1}^{\infty} \frac{a_{n,m}}{ ( x+m+n ) ^{\lambda}} \Biggr) \,dx \\ &=\frac{1}{\Gamma ( \lambda ) }\sum_{m=1}^{\infty}\sum _{n=1}^{\infty}a_{n,m} \int_{0}^{\infty }f(x) \biggl( \int_{0}^{\infty}t^{\lambda-1}e^{- ( m+n+x ) t}\,dt \biggr) \,dx\,dy \\ &=\frac{1}{\Gamma ( \lambda ) } \int_{0}^{\infty} \Biggl( t^{\frac{\lambda-1}{p}+\gamma}\sum _{m=1}^{\infty }\sum_{n=1}^{\infty}e^{-mt-nt}a_{n,m} \Biggr) \biggl( t^{\frac{\lambda-1}{q}-\gamma} \int_{0}^{\infty}e^{-tx}f(x)\,dx \biggr) \,dt \\ &\leq\frac{1}{\Gamma ( \lambda ) } \Biggl( \int_{0}^{\infty }t^{\lambda-1+p\gamma} \Biggl( \sum _{m=1}^{\infty }\sum_{n=1}^{\infty}e^{-mt-nt}a_{n,m} \Biggr) ^{p}\,dt \Biggr) ^{\frac{1}{p}} \\ &\quad\times \biggl( \int_{0}^{\infty}t^{\lambda-1-q\gamma} \biggl( \int_{0}^{\infty}e^{-tx}f(x)\,dx \biggr) ^{q}\,dt \biggr) ^{\frac{1}{q}}.\end{aligned} $$

By Lemmas 2.3 and 2.4 we get

$$\begin{aligned} J&\leq\frac{\Gamma(2-\theta q)^{\frac{1}{q}}\Gamma(1+\alpha p)^{\frac {1}{p}}}{\Gamma(\lambda) } \\ &\quad\times \Biggl( \int_{0}^{\infty}t^{\lambda +p\gamma+p\theta-2p+1} \Biggl( \sum _{m=1}^{\infty }\sum_{n=1}^{\infty}(m+n)^{p\theta}e^{-mt-nt}a_{n,m}^{p} \Biggr) \,dt \Biggr) ^{\frac{1}{p}} \\ &\quad\times \biggl( \int_{0}^{\infty}t^{\lambda-q\gamma-q\alpha -q} \int_{0}^{\infty}x^{-q\alpha}e^{-tx}f^{q}(x)\,dx\,dt \biggr) ^{\frac{1}{q}} \\ &=\tilde{C}_{\alpha, \theta} \Biggl( \sum_{m=1}^{\infty} \sum_{n=1}^{\infty }(m+n)^{2p-\lambda-p\gamma-2}a_{n,m}^{p} \Biggr) ^{\frac{1}{p}} \biggl( \int_{0}^{\infty}x^{q\gamma+q-\lambda-1}f^{q}(x)\,dx \biggr) ^{\frac{1}{q}},\end{aligned} $$

where

$$\tilde{C}_{\alpha, \theta}=\frac{\Gamma(2-\theta q)^{\frac {1}{q}}\Gamma(1+\alpha p)^{\frac{1}{p}}\Gamma ( \lambda+p\gamma +p\theta-2p+2 )^{\frac{1}{p}}\Gamma ( \lambda -q\gamma-q\alpha-q+1 )^{\frac{1}{q}}}{\Gamma(\lambda)}. $$

Finally, setting $\theta=\frac{2p-p\gamma-\lambda}{pq}$ and $\alpha =\frac{\lambda-q\gamma-q}{pq}$, we obtain inequality (16) with the constant $\tilde{C}_{\frac{\lambda-q\gamma-q}{pq}, \frac{2p-p\gamma-\lambda}{pq}}=C$. To prove that the constant *C* in (16) is the best possible, we define, for sufficiently small *ε*, the function $f_{\varepsilon}(x)=0$, on $( 0,1 ) $ and $f_{\varepsilon}(x)=x^{\frac{\lambda-\varepsilon}{q}-\gamma-1}$ on $[ 1,\infty ) $ and the sequence $\tilde{a}_{n,m}=(m+n)^{\frac {\lambda -\varepsilon}{q}+\gamma-2}$. If we assume that the constant *C* is not the best possible, then we may find $0<\tilde{K}<C$ such that

18 $$ \begin{aligned}[b] J&\leq\tilde{K} \Biggl( \sum_{m=1}^{\infty} \sum_{n=1}^{\infty }(m+n)^{2p-\lambda-p\gamma-2} \tilde{a}_{n,m}^{p} \Biggr) ^{\frac{1}{p}} \biggl( \int_{0}^{\infty}x^{q\gamma+q-\lambda-1}f_{\varepsilon }^{q}(x)\,dx \biggr)^{\frac{1}{q}} \\ &=\tilde{K} \Biggl( \sum_{m=1}^{\infty}\sum _{n=1}^{\infty }(m+n)^{-\varepsilon-2} \Biggr) ^{\frac{1}{p}} \biggl( \int_{1}^{\infty }x^{-\varepsilon-1}\,dx \biggr) ^{\frac{1}{q}} \\ &=\tilde{K} \Biggl( \sum_{m=1}^{\infty} \frac{1}{(m+1)^{2+\varepsilon}}+\sum_{n=2}^{\infty} \frac{1}{(n+1)^{2+\varepsilon}}+\sum_{m=2}^{\infty} \sum_{n=2}^{\infty}(m+n)^{-\varepsilon -2} \Biggr) ^{\frac{1}{p}} \biggl( \frac{1}{\varepsilon} \biggr) ^{\frac {1}{q}} \\ &\leq\tilde{K} \biggl( \int_{0}^{\infty}(x+1)^{-\varepsilon -2}\,dx+ \int_{1}^{\infty} ( 1+x ) ^{-\varepsilon -2}\,dx \\ &\qquad+ \int_{1}^{\infty} \int_{1}^{\infty}(x+y)^{-\varepsilon -2}\,dx\,dy \biggr) ^{\frac{1}{p}} \biggl( \frac{1}{\varepsilon} \biggr)^{\frac {1}{q}} \\ &=\tilde{K} \biggl( \frac{1}{\varepsilon+1}+\frac{1}{2^{\varepsilon +1} ( \varepsilon+1 ) }+\frac{1}{\varepsilon2^{\varepsilon }(1+\varepsilon)} \biggr) ^{\frac{1}{p}} \biggl( \frac{1}{\varepsilon} \biggr) ^{\frac {1}{q}} \\ &=\frac{\tilde{K}}{\varepsilon}L(\varepsilon).\end{aligned} $$

Here $L(\varepsilon)\rightarrow1$ as $\varepsilon\rightarrow0$. On the other hand, using similar computation as in (15), we have

19 $$ \begin{aligned}[b]J&= \int_{0}^{\infty}f_{\varepsilon}(x) \Biggl( \sum _{m=1}^{\infty}\sum_{n=1}^{\infty} \frac{\tilde{a}_{n,m}}{ ( x+m+n ) ^{\lambda}} \Biggr) \,dx \\ &= \int_{1}^{\infty}x^{\frac{\lambda-\varepsilon}{q}-\gamma-1} \Biggl( \sum _{m=1}^{\infty}\sum_{n=1}^{\infty} \frac{(m+n)^{\frac{\lambda -\varepsilon}{p}+\gamma-2}}{ ( x+m+n ) ^{\lambda}} \Biggr) \,dx \\ &\geq \int_{1}^{\infty}x^{\frac{\lambda-\varepsilon}{q}-\gamma -1} \biggl( \int_{1}^{\infty} \int_{1}^{\infty}\frac{(u+v)^{\frac{\lambda-\varepsilon}{p}+\gamma-2}}{ ( u+v+x )^{\lambda}}\,du\,dv \biggr)\,dx \\ &= \int_{1}^{\infty} \int_{1}^{\infty}(u+v)^{\frac{\lambda -\varepsilon}{p}+\gamma-2} \biggl( \int_{1}^{\infty}\frac{x^{\frac{\lambda-\varepsilon}{q}-\gamma-1}}{ ( u+v+x ) ^{\lambda}}\,dx \biggr) \,du\,dv \\ &=\frac{B ( \frac{\lambda-\varepsilon}{q}-\gamma,\frac{\lambda }{p}+\gamma+\frac{\varepsilon}{p} ) }{\varepsilon2^{\varepsilon }(1+\varepsilon)}-O(1).\end{aligned} $$

Clearly, as $\varepsilon\rightarrow0^{+}$, from (18) and (19) we obtain a contradiction. The proof of the theorem is completed. □

## Equivalent forms

In this section, we give some equivalent forms of the inequalities obtained in Theorems 3.1 and 3.2. All the inequalities are with the same best constant factor.

### Theorem 4.1

*Under the assumptions of Theorem* 3.1, *we have the following two inequalities*:

20 $$ \begin{aligned}[b] &\sum_{n=1}^{\infty}n^{(p-1)\lambda-\gamma p-1} \biggl( \int_{0}^{\infty} \int_{0}^{\infty}\frac{f(x,y)}{ ( x+y+n ) ^{\lambda}}\,dx\,dy \biggr) ^{p} \\ &\quad\leq C^{p} \int_{0}^{\infty } \int_{0}^{\infty} ( x+y ) ^{2p-\lambda-p\gamma -2}f^{p}(x,y)\,dx\,dy\end{aligned} $$

*and*

21 $$ \begin{aligned}[b]& \int_{0}^{\infty} \int_{0}^{\infty} ( x+y ) ^{ ( q-1 ) \lambda+\gamma q-2} \Biggl( \sum _{n=1}^{\infty}\frac{a_{n}}{ ( x+y+n ) ^{\lambda}} \Biggr) ^{q}\,dx\,dy \\ &\quad\leq C^{q}\sum_{n=1}^{\infty}n^{\gamma q-\lambda+q-1}a_{n}^{q}.\end{aligned} $$

*Both inequalities* (20) *and* (21) *are equivalent to* (12), *and the constants* $C^{p}$ *and* $C^{q}$ *are the best possible*.

### Proof

To prove (20), we set

$$ a_{n}=n^{(p-1)\lambda-\gamma p-1} \biggl( \int_{0}^{\infty } \int_{0}^{\infty}\frac{f(x,y)}{ ( x+y+n ) ^{\lambda}}\,dx\,dy \biggr) ^{p-1}. $$

Applying inequality (12), we find

22 $$ \begin{aligned}[b] &\sum_{n=1}^{\infty}n^{(p-1)\lambda-\gamma p-1} \biggl( \int_{0}^{\infty} \int_{0}^{\infty}\frac{f(x,y)}{ ( x+y+n ) ^{\lambda}}\,dx\,dy \biggr) ^{p} \\ &\quad=\sum_{n=1}^{\infty}n^{(p-1)\lambda-\gamma p-1} \biggl( \int_{0}^{\infty} \int_{0}^{\infty}\frac{f(x,y)}{ ( x+y+n ) ^{\lambda}}\,dx\,dy \biggr) ^{p-1} \\ &\qquad\times \biggl( \int_{0}^{\infty } \int_{0}^{\infty}\frac{f(x,y)}{ ( x+y+n ) ^{\lambda}}\,dx\,dy \biggr) \\ &\quad= \int_{0}^{\infty} \int_{0}^{\infty }\sum_{n=1}^{\infty} \frac{f(x,y)a_{n}}{ ( x+y+n ) ^{\lambda}}\,dx\,dy \\ &\quad\leq C \biggl( \int_{0}^{\infty} \int_{0}^{\infty} ( x+y ) ^{2p-\lambda-p\gamma-2}f^{p}(x,y)\,dx\,dy \biggr) ^{\frac{1}{p}} \Biggl( \sum_{n=1}^{\infty}n^{\gamma q+q-\lambda -1}a_{n}^{q} \Biggr) ^{\frac{1}{q}} \\ &\quad=C \biggl( \int_{0}^{\infty} \int_{0}^{\infty} ( x+y ) ^{2p-\lambda-p\gamma-2}f^{p}(x,y)\,dx\,dy \biggr) ^{\frac{1}{p}} \\ &\qquad\times \Biggl( \sum_{n=1}^{\infty}n^{(p-1)\lambda-\gamma p-1} \biggl( \int_{0}^{\infty} \int_{0}^{\infty}\frac{f(x,y)}{ ( x+y+n ) ^{\lambda}}\,dx\,dy \biggr) ^{p} \Biggr) ^{\frac{1}{q}}.\end{aligned} $$

Dividing both sides of inequality (22) by $( \sum_{m=1}^{\infty}n^{(p-1)\lambda-\gamma p-1} ( \int_{0}^{\infty}\int_{0}^{\infty}\frac{f(x,y)}{ ( x+y+n ) ^{\lambda}}\,dx\,dy ) ^{p} ) ^{\frac{1}{q}}$, we obtain inequality (20). On the other hand, by the Hölder inequality and (20), we find

$$\begin{aligned} & \int_{0}^{\infty} \int_{0}^{\infty}\sum_{n=1}^{\infty } \frac{f(x,y)a_{n}}{ ( x+y+n ) ^{\lambda}}\,dx\,dy \\ &\quad=\sum_{n=1}^{\infty} \biggl( n^{\frac{\lambda}{q}-\gamma-\frac {1}{p}} \int_{0}^{\infty} \int_{0}^{\infty}\frac{f(x,y)}{ ( x+y+n )^{\lambda}}\,dx\,dy \biggr) \bigl( n^{\frac{-\lambda }{q}+\gamma+\frac{1}{p}}a_{n} \bigr) \\ &\quad\leq \Biggl( \sum_{n=1}^{\infty}n^{(p-1)\lambda-\gamma p-1} \biggl( \int_{0}^{\infty} \int_{0}^{\infty}\frac{f(x,y)}{ ( x+y+n ) ^{\lambda}}\,dx\,dy \biggr)^{p} \Biggr)^{\frac{1}{p}} \\ &\qquad\times \Biggl( \sum_{n=1}^{\infty}n^{\gamma q+q-\lambda-1}a_{n}^{q} \Biggr) ^{\frac{1}{q}} \\ &\quad\leq \biggl( C^{p} \int_{0}^{\infty} \int_{0}^{\infty} ( x+y ) ^{2p-\lambda-p\gamma-2}f^{p}(x,y)\,dx\,dy \biggr)^{\frac {1}{p}} \\ &\qquad\times \Biggl( \sum_{n=1}^{\infty}n^{\gamma q+q-\lambda -1}a_{n}^{q} \Biggr) ^{\frac{1}{q}}. \end{aligned}$$

Therefore, using (20), we obtain (12).

Now, to prove the equivalence relation between (12) and (21), we set

$$ f(x,y)= ( x+y ) ^{ ( q-1 ) \lambda+\gamma q-2} \Biggl( \sum_{n=1}^{\infty} \frac{a_{n}}{ ( x+y+n ) ^{\lambda}} \Biggr) ^{q-1}. $$

By inequality (12) we get

$$\begin{aligned} & \int_{0}^{\infty} \int_{0}^{\infty} ( x+y ) ^{ ( q-1 ) \lambda+\gamma q-2} \Biggl( \sum _{n=1}^{\infty}\frac{a_{n}}{ ( x+y+n ) ^{\lambda}} \Biggr) ^{q}\,dx\,dy \\ &\quad= \int_{0}^{\infty} \int_{0}^{\infty} ( x+y ) ^{ ( q-1 ) \lambda+\gamma q-2} \Biggl( \sum _{n=1}^{\infty}\frac{a_{n}}{ ( x+y+n ) ^{\lambda}} \Biggr)^{q-1} \\ &\qquad\times \Biggl( \sum_{n=1}^{\infty} \frac{a_{n}}{ ( x+y+n ) ^{\lambda}} \Biggr) \,dx\,dy \\ &\quad= \int_{0}^{\infty} \int_{0}^{\infty }\sum_{n=1}^{\infty} \frac{f(x,y)a_{n}}{ ( x+y+n ) ^{\lambda}}\,dx\,dy \\ &\quad\leq C \biggl( \int_{0}^{\infty} \int_{0}^{\infty} ( x+y ) ^{2p-\lambda-p\gamma-2}f^{p}(x,y)\,dx\,dy \biggr) ^{\frac{1}{p}} \Biggl( \sum_{n=1}^{\infty}n^{\gamma q+q-\lambda -1}a_{n}^{q} \Biggr) ^{\frac{1}{q}} \\ &\quad=C \Biggl( \int_{0}^{\infty} \int_{0}^{\infty} ( x+y ) ^{ ( q-1 ) \lambda+\gamma q-2} \Biggl( \sum _{n=1}^{\infty}\frac{a_{n}}{ ( x+y+n ) ^{\lambda}} \Biggr)^{q}\,dx\,dy \Biggr)^{\frac{1}{p}} \\ &\qquad\times \Biggl( \sum_{n=1}^{\infty}n^{\gamma q+q-\lambda -1}a_{n}^{q} \Biggr) ^{\frac{1}{q}}. \end{aligned}$$

Obviously, from the last inequality we get (21). On the other hand, using the Hölder inequality and (21) respectively, we find

$$\begin{aligned} & \int_{0}^{\infty} \int_{0}^{\infty}\sum_{n=1}^{\infty } \frac{f(x,y)a_{n}}{ ( x+y+n )^{\lambda}}\,dx\,dy \\ &\quad= \int_{0}^{\infty} \int_{0}^{\infty} \bigl[ ( x+y )^{-\frac{ ( q-1 ) \lambda+\gamma q-2}{q}}f(x,y) \bigr] \\ &\qquad\times \Biggl[ ( x+y ) ^{\frac{ ( q-1 ) \lambda+\gamma q-2}{q}}\sum_{n=1}^{\infty}\frac{a_{n}}{ ( x+y+n ) ^{\lambda}} \Biggr] \,dx\,dy \\ &\quad\leq \biggl( \int_{0}^{\infty} \int_{0}^{\infty} ( x+y ) ^{2p-\lambda-p\gamma-2}f^{p}(x,y)\,dx\,dy \biggr) ^{\frac{1}{p}} \\ &\qquad\times \Biggl( \int_{0}^{\infty} \int_{0}^{\infty} ( x+y ) ^{ ( q-1 ) \lambda+\gamma q-2} \Biggl( \sum _{n=1}^{\infty}\frac{a_{n}}{ ( x+y+n ) ^{\lambda}} \Biggr) ^{q} \,dx\,dy\Biggr) ^{\frac {1}{q}} \\ &\quad\leq \biggl( \int_{0}^{\infty} \int_{0}^{\infty} ( x+y ) ^{2p-\lambda-p\gamma-2}f^{p}(x,y)\,dx\,dy \biggr) ^{\frac{1}{p}} \Biggl( C^{q}\sum _{n=1}^{\infty}n^{\gamma q+q-\lambda -1}a_{n}^{q} \Biggr) ^{\frac{1}{q}}.\end{aligned} $$

Thus, the equivalence relation between (21) and (12) is proved. Moreover, since the constant in (12) is the best possible, we deduce that the constants in both inequalities (20) and (21) are also the best possible. The theorem is proved. □

### Theorem 4.2

*Under the assumptions of the Theorem* 3.2, *we have the following inequalities*:

23 $$ \begin{aligned}[b] & \int_{0}^{\infty}x^{(p-1)\lambda-\gamma p-1} \Biggl( \sum _{m=1}^{\infty}\sum_{n=1}^{\infty} \frac{a_{m,n}}{ ( m+n+x ) ^{\lambda}} \Biggr) ^{p}\,dx \\ &\quad\leq C^{p}\sum_{m=1}^{\infty} \sum_{n=1}^{\infty} ( m+n ) ^{2p-\lambda-p\gamma-2}a_{m,n}^{p}\end{aligned} $$

*and*

24 $$ \begin{aligned}[b]&\sum_{m=1}^{\infty}\sum _{n=1}^{\infty} ( m+n ) ^{ ( q-1 ) \lambda+\gamma q-2} \biggl( \int_{0}^{\infty}\frac{f(x)}{ ( m+n+x ) ^{\lambda}}\,dx \biggr) ^{q}\,dx\,dy \\ &\quad\leq C^{q} \int_{0}^{\infty}x^{\gamma q-\lambda+q-1}f^{q}(x)\,dx.\end{aligned} $$

*Both inequalities* (23) *and* (24) *are equivalent to* (16), *and the constants* $C^{p}$ *and* $C^{q}$ *are the best possible*.

### Proof

To prove (23), we set

$$ f(x)=x^{(p-1)\lambda-\gamma p-1} \Biggl( \sum_{m=1}^{\infty } \sum_{n=1}^{\infty}\frac{a_{m,n}}{ ( m+n+x ) ^{\lambda}} \Biggr) ^{p-1}. $$

Applying inequality (16), we find

$$\begin{aligned} & \int_{0}^{\infty}x^{(p-1)\lambda-\gamma p-1} \Biggl( \sum _{m=1}^{\infty}\sum_{n=1}^{\infty} \frac{a_{m,n}}{ ( m+n+x ) ^{\lambda}} \Biggr) ^{p}\,dx \\ &\quad= \int_{0}^{\infty}x^{(p-1)\lambda-\gamma p-1} \Biggl( \sum _{m=1}^{\infty}\sum_{n=1}^{\infty} \frac{a_{m,n}}{ ( m+n+x ) ^{\lambda}} \Biggr) ^{p-1}\sum_{m=1}^{\infty } \sum_{n=1}^{\infty}\frac{a_{m,n}}{ ( m+n+x ) ^{\lambda}}\,dx \\ &\quad= \int_{0}^{\infty}\sum_{m=1}^{\infty } \sum_{n=1}^{\infty}\frac{f(x)a_{m,n}}{ ( m+n+x ) ^{\lambda}}\,dx \\ &\quad\leq C \Biggl( \sum_{m=1}^{\infty}\sum _{n=1}^{\infty} ( m+n ) ^{2p-\lambda-p\gamma-2}a_{n,m}^{p} \Biggr) ^{\frac {1}{p}} \biggl( \int_{0}^{\infty}x^{\gamma q+q-\lambda-1}f^{q}(x)\,dx \biggr) ^{\frac{1}{q}} \\ &\quad=C \Biggl( \sum_{m=1}^{\infty}\sum _{n=1}^{\infty} ( m+n ) ^{2p-\lambda-p\gamma-2}a_{n,m}^{p} \Biggr) ^{\frac{1}{p}} \\ &\qquad\times \Biggl( \int_{0}^{\infty}x^{(p-1)\lambda-\gamma p-1} \Biggl( \sum _{m=1}^{\infty}\sum_{n=1}^{\infty} \frac{a_{m,n}}{ ( m+n+x ) ^{\lambda}} \Biggr) ^{p}\,dx \Biggr) ^{\frac{1}{q}}.\end{aligned} $$

Hence, we obtain inequality (23). On the other hand, by the Hölder inequality and (23) we find

$$\begin{aligned} & \int_{0}^{\infty}f(x) \Biggl( \sum _{m=1}^{\infty }\sum_{n=1}^{\infty} \frac{a_{n,m}}{ ( x+y+n ) ^{\lambda}} \Biggr) \,dx \\ &\quad= \int_{0}^{\infty} \Biggl( x^{\frac{\lambda}{q}-\gamma-\frac{1}{p}}\sum _{m=1}^{\infty}\sum_{n=1}^{\infty} \frac{a_{m,n}}{ ( m+n+x ) ^{\lambda}} \Biggr) \bigl( x^{\frac {-\lambda}{q}+\gamma+\frac{1}{p}}f(x) \bigr) \,dx \\ &\quad\leq \Biggl( \int_{0}^{\infty}x^{(p-1)\lambda-\gamma p-1} \Biggl( \sum _{m=1}^{\infty}\sum_{n=1}^{\infty} \frac{a_{m,n}}{ ( m+n+x ) ^{\lambda}} \Biggr) ^{p}\,dx \Biggr)^{\frac{1}{p}} \biggl( \int_{0}^{\infty}x^{\gamma q+q-\lambda-1}f^{q}(x)\,dx \biggr) ^{\frac{1}{q}} \\ &\quad\leq \Biggl( C^{p}\sum_{m=1}^{\infty} \sum_{n=1}^{\infty } ( m+n ) ^{2p-\lambda-p\gamma-2}a_{m,n}^{p} \Biggr) ^{\frac {1}{p}} \biggl( \int_{0}^{\infty}x^{\gamma q+q-\lambda-1}f^{q}(x)\,dx \biggr) ^{\frac{1}{q}}.\end{aligned} $$

Therefore, using (23), we obtain (16). To prove the equivalence relation between (16) and (24), we set

$$ a_{m,n}= ( m+n ) ^{ ( q-1 ) \lambda+\gamma q-2} \biggl( \int_{0}^{\infty}\frac{f(x)}{ ( m+n+x ) ^{\lambda}}\,dx \biggr) ^{q-1}. $$

By inequality (16) we get

$$\begin{aligned} &\sum_{m=1}^{\infty}\sum _{n=1}^{\infty} ( m+n ) ^{ ( q-1 ) \lambda+\gamma q-2} \biggl( \int_{0}^{\infty}\frac{f(x)}{ ( m+n+x ) ^{\lambda}}\,dx \biggr)^{q} \\ &\quad=\sum_{m=1}^{\infty}\sum _{n=1}^{\infty} ( m+n ) ^{ ( q-1 ) \lambda+\gamma q-2} \biggl( \int_{0}^{\infty}\frac{f(x)}{ ( m+n+x )^{\lambda}}\,dx \biggr)^{q-1} \\ &\qquad\times \biggl( \int_{0}^{\infty}\frac{f(x)}{ ( m+n+x )^{\lambda}}\,dx \biggr) \\ &\quad= \int_{0}^{\infty}\sum_{m=1}^{\infty } \sum_{n=1}^{\infty}\frac{a_{m,n}f(x)}{ ( m+n+x ) ^{\lambda}}\,dx \\ &\quad\leq C \Biggl( \sum_{m=1}^{\infty}\sum _{n=1}^{\infty} ( m+n ) ^{2p-\lambda-p\gamma-2}a_{m,n}^{p} \Biggr) ^{\frac {1}{p}} \biggl( \int_{0}^{\infty}x^{\gamma q+q-\lambda-1}f^{q}(x)\,dx \biggr) ^{\frac{1}{q}} \\ &\quad=C \Biggl( \sum_{m=1}^{\infty}\sum _{n=1}^{\infty} ( m+n ) ^{ ( q-1 ) \lambda+\gamma q-2} \biggl( \int_{0}^{\infty}\frac{f(x)}{ ( m+n+x ) ^{\lambda}}\,dx \biggr) ^{q} \Biggr) ^{\frac{1}{p}} \\ &\qquad\times \biggl( \int_{0}^{\infty }x^{\gamma q+q-\lambda-1}f^{q}(x)\,dx \biggr) ^{\frac{1}{q}}. \end{aligned}$$

From the last inequality we obtain (24). On the other hand, using the Hölder inequality and (24), we have

$$\begin{aligned} & \int_{0}^{\infty}\sum_{m=1}^{\infty } \sum_{n=1}^{\infty}\frac{a_{m,n}f(x)}{ ( m+n+x ) ^{\lambda}}\,dx \\ &\quad=\sum_{m=1}^{\infty}\sum _{n=1}^{\infty} \bigl[ ( m+n )^{-\frac{ ( q-1 ) \lambda+\gamma q-2}{q}}a_{m,n} \bigr] \\ &\qquad\times \biggl[ ( m+n ) ^{\frac{ ( q-1 ) \lambda +\gamma q-2}{q}} \int_{0}^{\infty}\frac{f(x)}{ ( m+n+x )^{\lambda}}\,dx \biggr] \\ &\quad\leq \Biggl( \sum_{m=1}^{\infty}\sum _{n=1}^{\infty} ( m+n )^{2p-\lambda-p\gamma-2}a_{m,n}^{p} \Biggr)^{\frac{1}{p}} \\ &\qquad\times \Biggl( \sum_{m=1}^{\infty}\sum _{n=1}^{\infty} ( m+n ) ^{ ( q-1 ) \lambda+\gamma q-2} \biggl( \int_{0}^{\infty}\frac{f(x)}{ ( m+n+x ) ^{\lambda}}\,dx \biggr)^{q} \Biggr) ^{\frac {1}{q}} \\ &\quad\leq \Biggl( \sum_{m=1}^{\infty}\sum _{n=1}^{\infty} ( m+n ) ^{2p-\lambda-p\gamma-2}a_{m,n}^{p} \Biggr)^{\frac {1}{p}} \biggl( C^{q} \int_{0}^{\infty}x^{\gamma q+q-\lambda-1}f^{q}(x)\,dx \biggr) ^{\frac{1}{q}}.\end{aligned} $$

Thus, the equivalence relation between (24) and (16) is proved. Moreover, since the constant in (16) is the best possible, the constants in both inequalities (23) and (24) are also the best possible. The theorem is proved. □

## Conclusion

In the present study, we introduced two new half-discrete Hilbert inequalities for three variables. The equivalent forms are also considered. Moreover, we proved that the constants appearing on the right-hand sides of these inequalities are the best possible.
